# Prospective randomized controlled trial to compare laparoscopic distal gastrectomy (D2 lymphadenectomy plus complete mesogastrium excision, D2 + CME) with conventional D2 lymphadenectomy for locally advanced gastric adenocarcinoma: study protocol for a randomized controlled trial

**DOI:** 10.1186/s13063-018-2790-5

**Published:** 2018-08-09

**Authors:** Jie Shen, Beibei Cao, Yatao Wang, Aitang Xiao, Jichao Qin, Jianhong Wu, Qun Yan, Yuanlong Hu, Chuanyong Yang, Zhixin Cao, Junbo Hu, Ping Yin, Daxing Xie, Jianping Gong

**Affiliations:** 1 Department of GI Surgery, Tongji Hospital, Tongji Medical College, Huazhong University of Science and Technology, 1095 Jiefang Avenue, Wuhan, Hubei 430030 People’s Republic of China; 2 Department of Epidemiology and Biostatistics, School of Public Health, Tongji Medical College, Huazhong University of Science and Technology, Wuhan, 430030 People’s Republic of China

**Keywords:** Gastric cancer, Laparoscopic distal gastrectomy, D2 lymphadenectomy, Complete mesogastrium excision, Randomized controlled trial

## Abstract

**Background:**

Although radical gastrectomy with D2 lymph node dissection has become the standard surgical approach for locally advanced gastric cancer, patients still have a poor prognosis after operation. Previously, we proposed laparoscopic distal gastrectomy (D2 lymphadenectomy plus complete mesogastrium excision [D2 + CME]) as an optimized surgical procedure for locally advanced gastric cancer. By dissection along the boundary of the mesogastrium, D2 + CME resected proximal segments of the dorsal mesogastrium completely with less blood loss, and it improved the short-term surgical outcome. However, the oncologic therapeutic effect of D2 + CME has not yet been confirmed.

**Methods/design:**

A single-center, prospective, parallel-group, randomized controlled trial of laparoscopic distal gastrectomy with D2 + CME versus conventional D2 was conducted for patients with locally advanced gastric cancer at Tongji Hospital, Wuhan, China. In total, 336 patients who met the following eligibly criteria were included and were randomized to receive either the D2 + CME or D2 procedure: (1) pathologically proven adenocarcinoma; (2) 18 to 75 years old; cT2–4, N0–3, M0 at preoperative evaluation; (3) expected curative resection via laparoscopic distal gastrectomy; (4) no history of other cancer, chemotherapy, or radiotherapy; (5) no history of upper abdominal operation; and (6) perioperative American Society of Anesthesiologists class I, II, or III. The primary endpoint is 3 years of disease-free survival. The secondary endpoints are overall survival, recurrence pattern, mortality, morbidity, postoperative recovery course, and other parameters.

**Discussion:**

Previous studies have demonstrated the safety and feasibility of D2 + CME for locally advanced gastric cancer; however, there is still a lack of evidence to support its therapeutic effect. Thus, we performed this randomized trial to investigate whether D2 + CME can improve oncologic outcomes of patients with locally advanced gastric cancer. The findings from this trial may potentially optimize the surgical procedure and may improve the prognosis of patients with locally advanced gastric cancer.

**Trial registration:**

ClinicalTrials.gov, NCT01978444. Registered on October 31, 2013.

**Electronic supplementary material:**

The online version of this article (10.1186/s13063-018-2790-5) contains supplementary material, which is available to authorized users.

## Background

Gastric cancer is the main cause of cancer-related death in the world, especially in East Asia [1]. Although substantial improvements have been achieved in diagnosis and treatment, patients with advanced gastric cancer (AGC) still have a poor prognosis [2]. Surgery is the only curative option for locally advanced gastric cancer. According to the Japanese gastric cancer treatment guidelines [3], gastrectomy with extended (D2) lymphadenectomy is the standard approach in the surgical treatment of gastric cancer. Even so, the recurrence rate of patients with locally advanced gastric cancer who undergo radical operation is still approximately 60% [4].

Dissemination of neoplastic cells has been proven to be the main reason for tumor relapse and cancer-related mortality [5]. To avoid tumor cell spreading or to minimize the residual during operation, *en bloc* resection of the primary lesion and its adjacent tissues, such as the mesentery of the gastrointestinal tract, has begun to be the gold standard of radical surgery [6]. In colon and rectal cancers, complete mesocolic excision and total mesorectal excision have been widely used, and they have improved prognosis significantly [7–11]. However, there is no such operation in the stomach. Conventional D2 lymphadenectomy, which is performed by looking for blood vessels in adipose or connective tissues and by dissecting lymph nodes individually, is still the mainstream surgical procedure for gastric cancer.

Our previous studies have demonstrated the existence of disseminated cancer cells (named metastasis V) in the mesogastrium [12, 13] that presented an understandable mesogastrium model for gastrectomy [14]. Therefore, we put forward D2 lymphadenectomy plus complete mesogastrium excision (D2 + CME) to resect both the primary lesion and adjacent tissue as completely as possible to avoid residual tumor or cancer cell spreading [15]. Although a retrospective study has shown that D2 + CME exhibited advantages in intraoperative hemorrhage and postoperative recovery course [15], there is still no prospective randomized controlled trial assessing its therapeutic effect. To further evaluate the D2 + CME procedure, we initiated this single-center, prospective, randomized controlled trial to compare disease-free survival (DFS), overall survival (OS), recurrence, mortality, morbidity, postoperative recovery course, and other parameters between D2 + CME and the conventional D2 procedure in patients with locally advanced gastric cancer who underwent laparoscopic distal gastrectomy.

### Rationale for the trial

Radical gastrectomy with D2 lymphadenectomy has been the standard surgical procedure for AGC and has significantly improved patient outcome. Even so, patients with locally advanced gastric cancer still have a high recurrence rate and poor prognosis. The main causes of postoperative recurrence might be the minimal residual or potential cancer cell dissemination during operation. Thus, resection of both the primary tumor and the adjacent mesentery as completely as possible to avoid minimal residual or cancer cell spreading should become the optimal surgical procedure.

Previously, we presented the mesogastrium model for gastrectomy and developed an optimal surgical approach, D2 + CME, to achieve a complete excision of the gastric mesentery. In our retrospective study, we demonstrated that this approach showed several advantages in terms of decreasing intraoperative bleeding (12.44 ± 22.89 ml, range 5–100), improving lymph node harvesting (35.04 ± 10.70, range 14–55), and shortening the postoperative recovery course (11.09 ± 4.28 days, range 8–28). However, there is still a lack of evidence derived from a prospective randomized controlled trial. Thus, we conducted this study to evaluate the 3-year DFS, 3-year OS, recurrence, mortality, morbidity, postoperative recovery course, and other outcomes of laparoscopic distal gastrectomy with D2 + CME for locally advanced gastric cancer. In summary, the main purpose of this study is to evaluate the therapeutic results of the D2 + CME procedure and to provide evidence for this optimal surgical approach.

## Methods/design

### Study aims and objectives

The key aim of this study is to assess whether D2 + CME is superior to conventional D2 lymphadenectomy in terms of 3-year DFS. The secondary objectives are to compare D2 + CME with conventional D2 in terms of mortality, morbidity, operative time, intraoperative bleeding mount, harvested lymph node number, compliance rates, and quality of life.

### Trial design

This is a prospective, controlled, randomized single-center trial evaluating the therapeutic effect of laparoscopic distal gastrectomy with D2 + CME versus conventional D2 in 336 patients with gastric cancer. Randomization is at the patient level. Participants are randomized 1:1 to receive either D2 + CME or D2.

This study is designed to evaluate the superiority of D2 + CME compared with D2 alone in terms of DFS. We are supposing differences in 3-year DFS of no less than 15% (60% in D2 vs. 75% in D2 + CME) as demonstrating superiority.

**Fig. 1:**
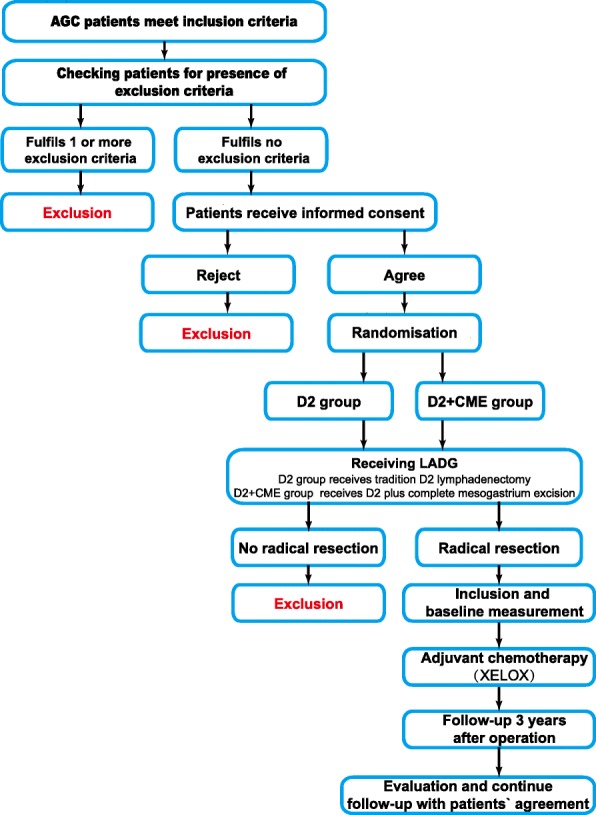
Study flow diagram. *AGC* Advanced gastric cancer, *D2* Gastrectomy with D2 lymphadenectomy, *D2 + CME* D2 lymphadenectomy plus complete mesogastrium excision, *LADG* Laparoscopy-assisted distal gastrectomy, *XELOX* Chemotherapy regimen consisting of capecitabine combined with oxaliplatin

Patients enrolled in this trial are asked to complete at least 3 years of follow-up after operation. Then, with allowance, follow-up will last for 5 years. Follow-up is at 3-month intervals for the first 2 years and at 6-month intervals for the last 3 years. At every follow-up, the patients will undergo a physical examination; their nutritional status will be assessed; and blood tests (including blood count, liver function test, tumor markers) will be performed. Abdominal computed tomography (CT) and endoscopy will be performed twice annually. The date and site of the first recurrence, as well as the date of death, will be recorded. The study flow diagram is shown in Fig. 1, and the Standard Protocol Items: Recommendations for Interventional Trials (SPIRIT) checklist is provided in Additional file 1.

### Setting

Participants have been recruited and operated at the Department of Gastrointestinal Surgery, Tongji Hospital, Wuhan, China. There are approximately 350 cases of laparoscopic radical gastrectomy in the hospital per year.

### Study duration

Recruitment for this study began in September 2014 and was completed in June 2018. The 3-year follow-up for all the participants will be completed in the middle of 2021.

### Participants

Patients with AGC who meet the following criteria will be recruited into this study.

#### Inclusion criteria

1. Aged older than 18 years and younger than 75 years
2. Body mass index less than 30 kg/m^2^
3. Primary gastric adenocarcinoma confirmed pathologically by endoscopic biopsy
4. cT2–4, N0–3, M0 at preoperative evaluation according to the *AJCC Cancer Staging Manual, Seventh Edition* [16]
5. Expected curative resection via laparoscopic distal gastrectomy
6. Eastern Cooperative Oncology Group Performance Status 0 or 1 and American Society of Anesthesiologists (ASA) class I, II, or III
7. Written informed consent

#### Exclusion criteria

1. Pregnant or breastfeeding women
2. Severe mental disorder
3. Previous neoadjuvant chemotherapy or radiotherapy
4. Previous upper abdominal surgery
5. Other malignant diseases or combined with another gastric malignant tumor (including lymphoma and gastric stromal tumor)
6. Total gastrectomy

#### Selection of gastrectomy

The selection of gastrectomy is according to the Japanese gastric cancer treatment guidelines (2010 version 3) [3]. Briefly, distal gastrectomy is performed when a satisfactory proximal resection margin (at least 3 cm for T2 or deeper tumors with an expansive growth pattern and 5 cm for those with infiltrative growth pattern) can be obtained. Otherwise, total gastrectomy should be considered. The extent of gastrectomy is evaluated by abdominal CT and laparoscopic exploration. Due to the aim of our research, patients who are not suitable for distal gastrectomy will be excluded.

### Interventions

#### Operative approach

**Fig. 2:**
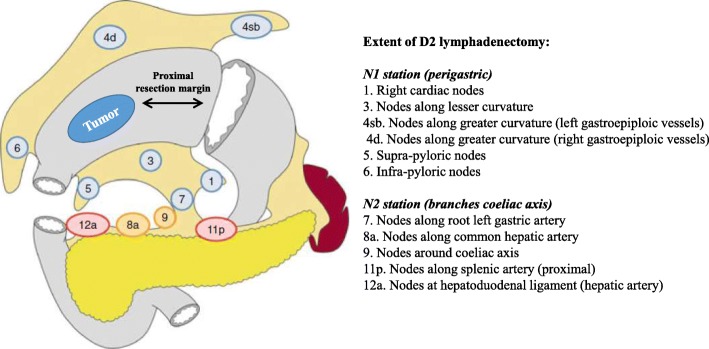
Gastrectomy and lymph node dissection in the conventional D2 procedure. The proximal margin of gastrectomy should achieve at least 3 cm for T2 or deeper tumors with an expansive growth pattern or 5 cm for those with an infiltrative growth pattern. The lymphadenectomy should include 1, 3, 4sb, 4d, 5, 6, 7, 8a, 9, 11p, and 12a groups of lymph nodes [3]

Patients in the D2 group receive laparoscopy-assisted distal gastrectomy (LADG) with conventional D2 lymphadenectomy according to the Japanese gastric cancer treatment guidelines (2010, version 3). Operations are performed routinely by experienced surgeons who do not receive training for the D2 + CME procedure. The extent of lymphadenectomy includes Nos. 1, 3, 4sb, 4d, 5, 6, 7, 8a, 9, 11p, and 12a. Dissection of No. 14v is optional, and omentectomy is necessary. Reconstruction is performed in the standard Billroth I/II or Roux-en-Y fashion. The type of reconstruction is determined by the surgeon’s experience (Fig. 2).

Patients in the D2 + CME group receive standardized LADG with D2 + CME [15], which is performed by the founder of this procedure. The standard D2 + CME procedure should meet the following criteria:

1. Clearly exposing five mesogastrium (left gastroepiploic mesentery, right gastroepiploic mesentery, left gastric mesentery, right gastric mesentery, and postgastric mesentery)
2. *En bloc* separation of the mesentery from the mesenteric bed
3. Dissecting along the root of the mesentery
4. Ligation should reach the root of the blood vessels.
5. After the mesentery is dissected, the lower side of the mesogastrium should be flat and smooth.

**Fig. 3:**
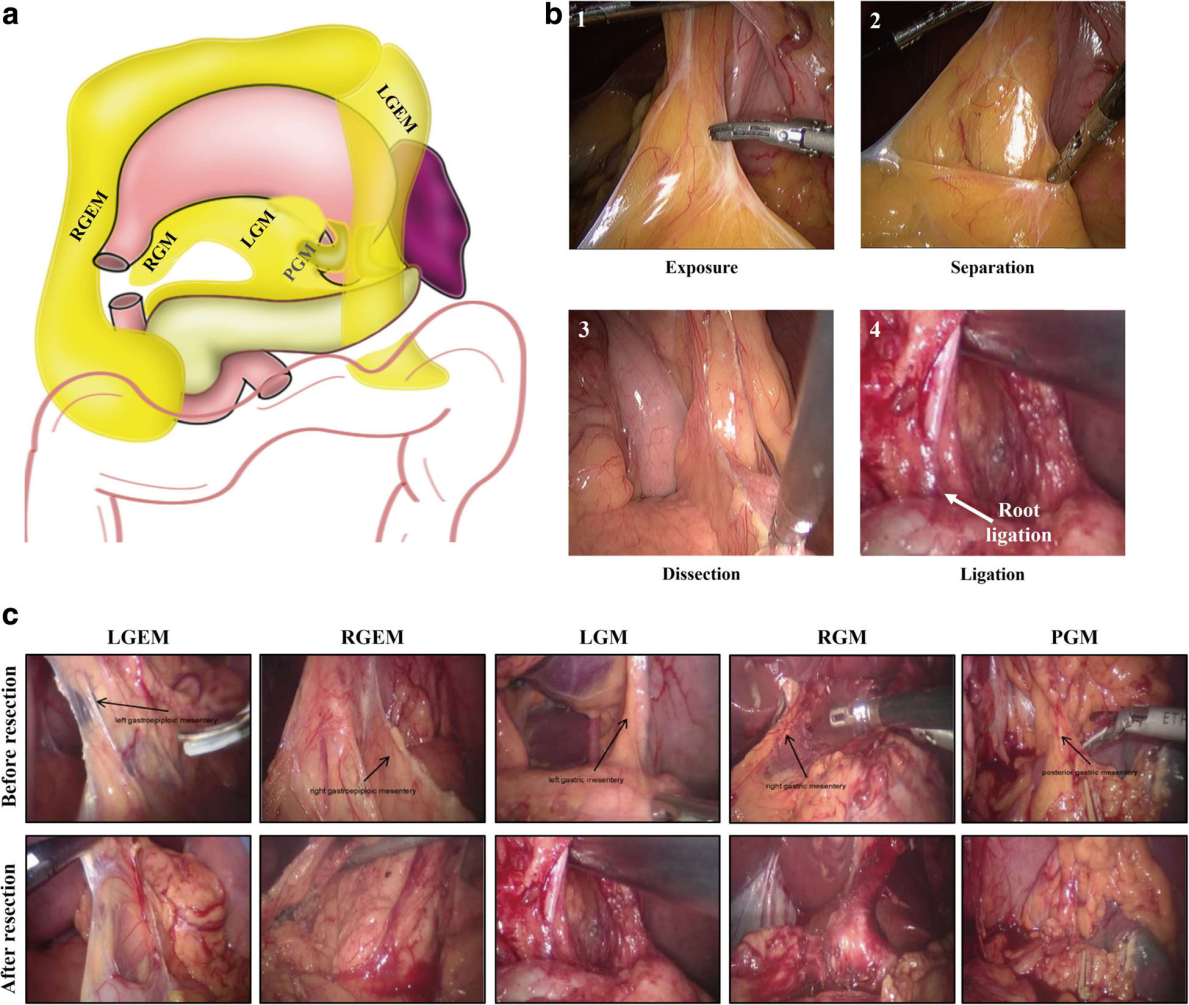
**a** Diagram of resected mesogastrium (*yellow*) during D2 + CME. **b** Intraoperative photographs show the standard procedures of mesenteric excision: (**1**) expose the mesogastrium clearly, (**2**) separate the mesentery from the mesenteric bed, (**3**) dissect along with the root of the mesentery, and (**4**) ligation should reach the root of the blood vessels. **c** Pictures of each mesogastrium were photographed under laparoscopy before (*left*) and after dissection (*right*) during D2 + CME. *LGEM* Left gastroepiploic mesentery, *RGEM* Right gastroepiploic mesentery, *LGM* Left gastric mesentery, *RGM* Right gastric mesentery, *PGM* Postgastric mesentery. *Black arrow* = mesogastrium [14]l

Before and after mesentery dissection, two photographs will be taken individually in each mesogastric area to evaluate the integrity of the mesenteric excision (Fig. 3).

#### Postoperative management and adjuvant chemotherapy

Gastrointestinal function is evaluated twice per day; the gastric canal will be pulled out, and a liquid diet will be suggested beginning after the first flatus is confirmed. If patients are fully tolerant of a liquid diet for 4 days, they can begin a soft diet. Adjuvant chemotherapy will be started at the fourth or fifth week after surgery without contraindication [17].

All the patients in this trial are recommended to receive adjuvant chemotherapy. The first-line regimen is XELOX (oxaliplatin 130 mg/m^2^ on day 1 and capecitabine 1000 mg/m^2^ twice daily on days 1–14 of a 3-week cycle for eight cycles in 6 months). After each cycle, the toxicity of chemotherapy is graded according to the Nation Cancer Institute’s Common Terminology Criteria for Adverse Events (version 4) [18]. If grade 3 or 4 toxicity occurs, chemotherapy will be delayed up to 3 weeks, until the patient recovers from the adverse effects. When a patient experiences serious side effects and cannot tolerate the treatment, the drug dose should be reduced or chemotherapy could be stopped completely. Once a patient’s chemotherapy regimen is changed, the reason should be recorded.

### Surgeons and surgical quality control

Surgeons who will participate in this study will meet the following criteria: (1) have performed at least 100 cases of laparoscopic distal gastrectomy with D2 lymphadenectomy and (2) have performed at least 50 gastrectomies annually. These criteria qualified eight surgeons to participate in this study. Among them, seven surgeons who have had no training in the D2 + CME procedure are assigned to the D2 group, and the remaining one, who is the founder of the D2 + CME procedure, performs operations in the D2 + CME group.

Surgical quality control is evaluated by using intraoperative video recordings and the resection margin of the specimens. The extent of gastrectomy and lymph node dissection is based on the Japanese gastric cancer treatment guidelines (2010, version 3) [3]. In the D2 group, the intraoperative videos should clearly show the lymph dissection of stations including 1, 3, 4sb, 4d, 5, 6, 7, 8a, 9, 11p, and 12a. Furthermore, the dissected specimen should be checked by an experienced surgeon or a pathologist to ensure that the proximal resection margin and the removal of lymph nodes are satisfactory (Additional file 2). In the D2 + CME group, under the premise of meeting D2 qualification, the operative procedure should also be under the quality control of CME. Quality control of CME is based mainly on the intraoperative video recordings. In the D2 + CME procedure, the resection of the left and right mesogastria, the left and right gastroepiploic mesogastria, and the posterior mesogastrium should be clearly shown (Fig. 3). Furthermore, a mesenteric scoring system that includes the following parameters is applied for each of mesenteric region: trijunction point exposure (exposure of the incision of the mesentery), mesogastrium body (smooth and shiny surface of mesentery after resection), smooth plane of the surgical bed after mesenteric resection, and high tie ligation of the vessel. According to the quality of surgery, each parameter is scored as 2 (good), 1 (moderate), or 0 (poor) (Additional file 3). Then, the parameter scores are summed to get the mesenteric score. Considering that the posterior mesogastrium is short and often lacks blood supply, we simply score it as 2, 1, or 0. The scores of the operation should be recorded in a formal table (Additional file 4) and analyzed. In a standard D2 + CME surgery, the sum of the mesenteric scores should be at least 30.

### Withdrawal

All the patients are freely informed to participate in this study and can decide to withdraw from this trial at any time. If a patient withdraws, his/her information will not be recorded in this study. However, the research team can still collect outcome data from the healthcare records. Patients provide informed consent that their personal data held by this study can be freely withdrawn; otherwise, these data will be retained.

### Outcome measures

#### Primary endpoint

Three-year DFS will be evaluated in 2021. Once a patient dies, the death date, cause of death, and how the death was identified will be entered on the case report form (CRF). Once a subject is lost after the last follow-up, the patient’s survival data will be collected by communicating with the patient or with his/her family via telephone or letter.

#### Secondary endpoints

1. *Recurrence patterns*: Recurrence will be assessed by routine follow-up. The recurrence location and time will be recorded when diagnosed. The recurrence date, site, and pattern, as well as how the recurrence is diagnosed, are recorded on the CRF. The recurrence pattern is classified into six categories: locoregional, lymphatic, peritoneal, hematogenous, mixed, and unclassified. Locoregional recurrence includes tumor recurrence in the anastomosis, gastric stump, and adjacent tissue, including gastric bed, porta hepatis, regional lymph nodes (perigastric, left gastric, common hepatic, celiac, and hepatoduodenal), as well as the adjacent abdominal wall. Lymphatic recurrence includes tumor recurrence in distant lymph nodes, including para-aortic, inguinal, postperitoneal, Virchow’s nodes, and other distant sites. Peritoneal recurrence is defined as peritoneal seeding or Krukenberg tumor. Hematogenous recurrence means that the tumor relapses in the liver, lung, bone, brain, or other distant organs. A mixed pattern includes those recurrences that meet more than one of the above categories. Suspected recurrence, which lacks imaging or endoscopy evidence, falls in the unclassified recurrence pattern category.
2. *Morbidity and mortality*: Postoperative morbidity and mortality are evaluated within 30 days after operation. All the complications are diagnosed on the basis of either clinical examination or symptoms. Anastomosis-related complications, such as anastomosis bleeding, leakage, and stenosis, will be confirmed by endoscopy, abdominal CT, gastrointestinal X-ray imaging, or angiography. Abdominal abscesses will be proven by ultrasonography or a CT scan that reveals the presence of septic fluid in the abdominal cavity, with the patient’s temperature higher than 38 °C for more than 24 hours. Intraoperative and postoperative hemorrhage will be defined as an amount of bleeding more than 300 ml. Pancreatic injury will be diagnosed by an increase in serum amylase level more than three times the upper limit of normal and obvious clinical symptoms. Lymphatic leakage will be confirmed by a positive chyle test when the patient’s abdominal drainage fluid is more than 300 ml/d for 5 continuous days after operation. Intestinal fistula will be confirmed by the presence of a bowel-to-bowel or a bowel-to-cutaneous fistula tract via fistulogram. Intestinal obstruction will be diagnosed by the lack of a bowel movement for more than 5 days after surgery; a simple x-ray examination will show mechanical obstruction with air-fluid level or paralytic ileus. Other complications, such as diarrhea, lymphorrhea, urinary tract morbidities, and respiratory morbidities, should be diagnosed by corresponding clinical symptoms and examinations. The severity of postoperative complications will be assessed according to the Clavien-Dindo classification [19]. In addition, 90-day postoperative mortality data will also be collected and analyzed to evaluate the short-term outcome of the operation.
3. *Intraoperative complications*: Intraoperative complications will be evaluated during operation. Intraoperative complications will be defined as hemorrhage caused by named vascular injury, visceral organ injury, mechanical factor-related injury, cardiopulmonary dysfunction due to hypercapnia, and others.
4. *Cost-effectiveness*: The total in-hospital cost will be assessed. The two groups will be compared with regard to cost-effectiveness. Information related to cost (e.g., length of hospital stay, complications, comorbidity) will be collected and evaluated.

**Fig. 4:**
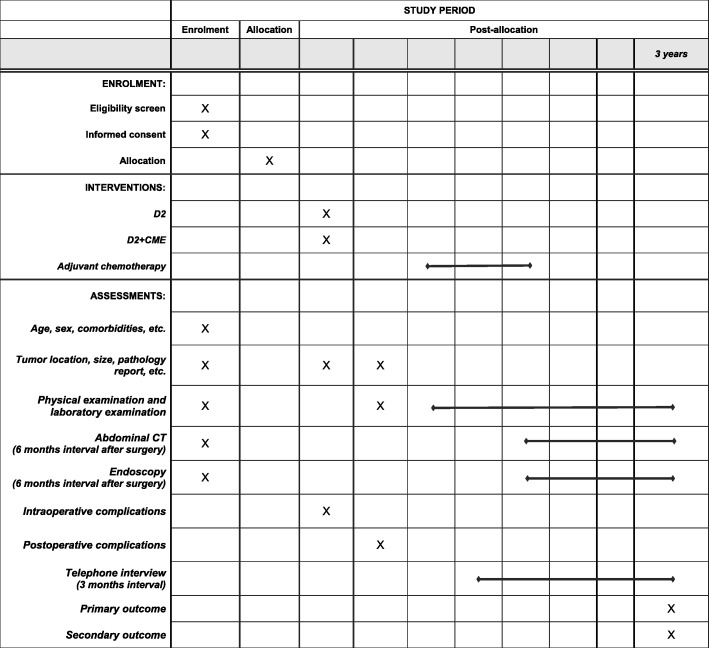
Schedule of enrollments, interventions, and assessments

The timing of our enrollment, intervention, and outcome measurements are summarized in Fig. 4.

### Sample size

This study was designed to evaluate the superiority of D2 + CME compared with D2 alone in terms of DFS. The sample size is calculated through two independent proportions power analysis. The calculated sample size is 304 (152 per group), with a two-sided α of 5% and an 80% statistical power (z-test) to detect a supposed 3-year DFS difference of 15% (60% in D2 group vs. 75% in D2 + CME group). Considering potential withdrawals, an additional 10% of patients will be investigated to guarantee that the final sample size is large enough. Therefore, the planned sample size is 336 (168 per group). The enrollment period will last for 3 years.

### Randomization, masking, and data collection

All the eligible patients will be registered in this study and randomized to either the D2 + CME group or the D2 group (1:1) on the basis of a computer-generated randomization list (generated using SAS version 9.2 software; SAS Institute, Cary, NC, USA) with a block size of 6. Patient enrollment and randomization will be performed by an independent data collection group (managed by the Department of Epidemiology and Biostatistics, Huazhong University of Science and Technology). The data collection group will then collect patient information and fill out the CRF, including general characteristics, preoperative staging, operative findings, pathological reports, and postoperative outcomes. All the data will be placed into the local database via a data registry server managed by the data collection group. In this trial, the patients and the follow-up staff are masked to the treatment allocation.

### Statistical analysis

A statistical analysis plan will be developed and agreed upon by the data collection group. In summary, the continuous data will be presented as the mean ± SD and will be statistically analyzed with Student’s *t* test or the Wilcoxon rank-sum test. The categorical variables will be compared between the D2 and D2 + CME groups by the χ^2^ test or Fisher’s exact test. Grade data, including age, ASA score, and Clavien-Dindo classification, will be compared with the Wilcoxon signed-rank test. The survival curves for 3-year DFS (primary endpoints) and OS will be estimated via the Kaplan-Meier method and will be compared by log-rank test. All the *p* values are based on two-sided statistical tests; *p* < 0.05 is considered statistically significant.

Two interim analyses will be planned in this trial. The first interim analysis will be planned for the date at which half of the planned sample size has been enrolled to assess the safety of this study. The second will be held when the entire planned sample size has been enrolled. The stopping criteria in the study protocol are as follows:

1. Complication rate and mortality in the D2 + CME group are significantly higher than those in the D2 group, leading to study termination owing to the unsafety of the CME procedure

2. Survival in the D2 + CME group is significantly superior to that of the D2 group, leading to study termination owing to the efficacy of the CME procedure

3. Survival in the D2 + CME group is significantly poorer than that of the D2 group, leading to study termination owing to futility of the CME procedure

### Interim analysis

Our first interim analysis was completed on September 23, 2016. In this analysis, a total of 165 patients were recruited and randomized (D2 + CME, 82 patients; D2, 83 patients). There were no significant differences in age, sex, comorbidities, and ASA scores. The D2 + CME procedure exhibited an advantage in intralaparoscopic bleeding (23.87 ± 19.27 vs. 39.72 ± 32.28, *p* < 0.01) and lymph node harvesting (34.31 ± 11.80 vs. 25.73 ± 10.83, *p* < 0.01). The postoperative morbidity of the D2 + CME and D2 groups were 13.4% (11 of 82) and 18.1% (15 of 83), respectively (*p* = 0.412). Reoperation was required in only one case in the D2 group. The postoperative mortality was 0% in both groups. These results indicate that there is no significant difference in the complications between the D2 + CME group and the conventional D2 group. Therefore, we have ensured that this trial is safe, and thus it is ongoing.

## Discussion

In gastrointestinal carcinoma, the prognoses of colon and rectal cancers are significantly improved due to the wide use of complete mesenteric excision [7–11]. However, in gastric cancer, the concept of mesogastric excision has not been widely recognized. In our previous study, we proposed the structure and classification of mesogastrium [14], and we put forward a new surgical approach, D2 + CME, to achieve a complete excision of gastric mesentery based on D2 lymphadenectomy [15]. Compared with conventional D2 lymphadenectomy, D2 + CME places more emphasis on dissection along the “boundary” of the mesentery rather than the surgical plane, and it removes the primary lesion and the adjacent soft tissue completely to avoid potential tumor dissemination and remnants.

Theoretically, D2 + CME decreases the incidence of intraoperative cancer dissemination and tumor residuals in the mesogastrium. Furthermore, dissection along the mesenteric boundary conforms to the anatomical structure of the stomach and leads to less intraoperative bleeding. In clinical practice, D2 + CME has been demonstrated to be safe and maneuverable. In our retrospective study, we confirmed that this procedure can achieve D2 lymph node dissection while reducing intraoperative hemorrhage [15]. Patients undergoing the D2 + CME procedure appear to have a shorter postoperative recovery course and to have fewer complications [15].

Although we have obtained evidence supporting the short-term efficacy of D2 + CME for locally advanced gastric cancer, there is little information about its oncologic outcomes. Thus, we performed this trial to evaluate the therapeutic effect of D2 + CME. D2 + CME is an innovative procedure in the surgical treatment of gastric cancer and has not been widely popularized. For the purpose of avoiding potential risks, our current research focuses on patients who underwent laparoscopic distal gastrectomy. Recruitment in this study began in September 2014, and the interim analysis was performed on September 23, 2016. In this analysis, there was no significant difference in the postoperative morbidity and mortality between the two groups. In addition, D2 + CME exhibited advantages in intralaparoscopic bleeding and lymph node harvesting. Through this interim evaluation, D2 + CME has been partially demonstrated to be safe and feasible in the treatment of locally advanced gastric cancer. For the sake of further evaluation of the efficiency and effectiveness of this procedure, our study is ongoing.

The aim of this trial is to evaluate the D2 + CME procedure in patients with locally advanced gastric cancer who underwent laparoscopic distal gastrectomy. Distal gastrectomy was performed when a satisfactory proximal resection margin could be achieved. Although histologic subtypes of gastric cancer have been reported to be potential risk factors for tumor invasiveness and prognosis [20], the selection of the type of operation does not depend on the histologic subtype. In addition, patients with metastatic gastric cancer were excluded from our research. Although some previous studies have proven that operation plus neoadjuvant chemotherapy showed a favorable survival for limited metastatic gastric cancer [21, 22], surgical treatment for metastatic gastric cancer is still controversial. The REGATTA study, a multicenter randomized controlled trial focusing on the treatment strategy for patients with stage IV gastric cancer, demonstrated that surgical treatment did not show any survival benefit in AGC with a single noncurable factor [23]. Moreover, in the National Comprehensive Cancer Network, European Society for Medical Oncology, and Japanese Gastric Cancer Association guidelines, chemotherapy is still the main approach for treating metastatic gastric cancer [3, 24, 25]. Therefore, a surgical approach is not the first recommendation for patients with stage IV AGC in our institution.

In summary, as an innovative surgical procedure, D2 + CME still needs more clinical trials to assess its effectiveness in locally advanced gastric cancer. This study is the first single-center randomized controlled trial that will investigate whether D2 + CME can improve oncologic outcomes of patients with locally advanced gastric cancer. The findings from this trial may potentially optimize the surgical procedure and may improve the prognosis of patients with locally advanced gastric cancer.

## Trial status

This trial began recruitment in September 2014, with the first participant enrolled on 22 September 2014. Enrollment was completed on 28 June 2018.

## Additional files

Additional file 1: Standard Protocol Items: Recommendations for Interventional Trials (SPIRIT) checklist. (DOC 124 kb)

Additional file 2: Photograph of the dissected specimen after operation. Removal of lymph nodes should include the 1, 3, 4sb, 4d, 5, 6, 7, 8a, 9, 11p, and 12a groups, and the proximal resection margin should achieve at least 3 cm for T2 or deeper tumors with an expansive growth pattern and 5 cm for those with an infiltrative growth pattern. (PDF 600 kb)

Additional file 3: Instance of the scoring criterion for mesenteric excision in D2 + CME procedure. Mesenteric scoring including four parameters: trijunction point exposure, mesogastrium body, smooth plane of surgical bed after mesenteric resection, and the high tie ligation of vessels. Each parameter is scored as 2 (good), 1 (moderate), or 0 (poor). The parametric scores are summed to get the mesenteric score, then the mesenteric scores of all the dissected mesogastria are summed to get the total score. (PDF 179 kb)

Additional file 4: Record table of mesenteric scoring in the D2 + CME procedure. (PDF 260 kb)

## Abbreviations

AGC: Advanced gastric cancer
ASA: American Society of Anesthesiologists
CME: Complete mesogastrium excision
CRF: Case report form
CT: Computed tomography
D2: Gastrectomy with extended (D2) lymphadenectomy
D2 + CME: D2 Lymphadenectomy plus complete mesogastrium excision
DFS: Disease-free survival
LADG: Laparoscopy-assisted distal gastrectomy
LGEM: Left gastroepiploic mesentery
LGM: Left gastric mesentery
OS: Overall survival
PGM: Postgastric mesentery
RGEM: Right gastroepiploic mesentery
RGM: Right gastric mesentery
XELOX: Chemotherapy regimen consisting of capecitabine combined with oxaliplatin
