# Supplementary material for: Prospective randomized controlled trial to compare laparoscopic distal gastrectomy (D2 lymphadenectomy plus complete mesogastrium excision, D2 + CME) with conventional D2 lymphadenectomy for locally advanced gastric adenocarcinoma: study protocol for a randomized controlled trial
Source: Trials. 2018 Aug 9;19:432. doi: 10.1186/s13063-018-2790-5 (PMC6085680; doi:10.1186/s13063-018-2790-5)
Supplement: Supplementary file 2 — Photograph of the dissected specimen after operation. Removal of lymph nodes should include the 1, 3, 4sb, 4d, 5, 6, 7, 8a, 9, 11p, and 12a groups, and the proximal resection margin should achieve at least 3 cm for T2 or deeper tumors with an expansive growth pattern and 5 cm for those with an infiltrative growth pattern. (PDF 600 kb) [file 13063_2018_2790_MOESM2_ESM.pdf]

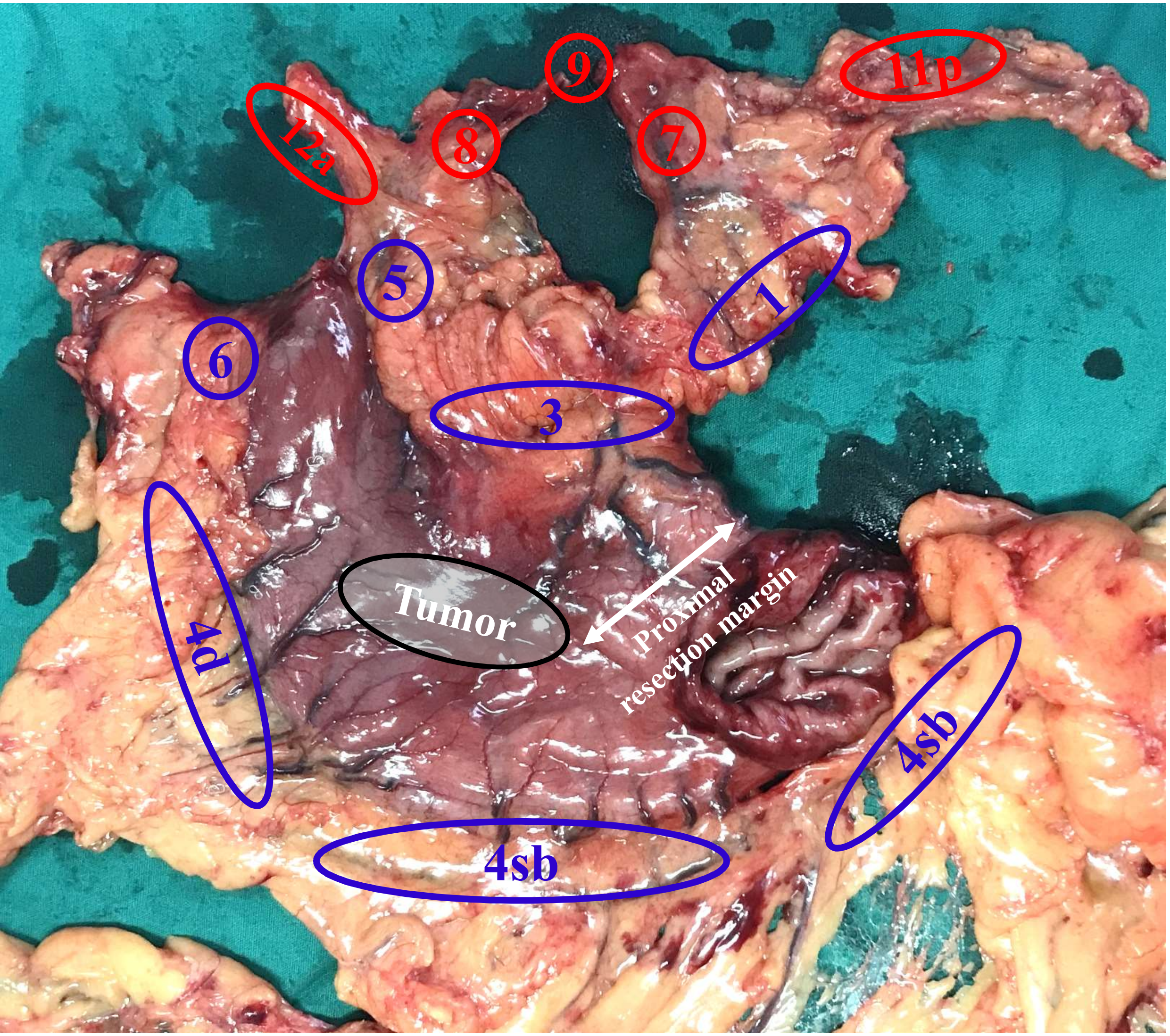

Proximal resection margin

At least 3 cm for T2 or deeper tumors with an expansive growth pattern;

At least 5 cm for those with infiltrative growth pattern.

Removal lymph nodes:

N1 station (perigastric)

1. Right cardiac nodes
3. Nodes along lesser curvature

4sb. Nodes along greater curvature (left gastroepiploic vessels)

4d. Nodes along greater curvature (right gastroepiploic vessels)

5. Supra-pyloric nodes
6. Infra-pyloric nodes

N2 station (branches coeliac axis)

7. Nodes along root left gastric artery
8. Nodes along common hepatic artery
9. Nodes around coeliac axis

11p. Nodes along splenic artery (proximal)

12a. Nodes at hepatoduodenal ligament (hepatic artery)
