# Supplementary material for: Prospective randomized controlled trial to compare laparoscopic distal gastrectomy (D2 lymphadenectomy plus complete mesogastrium excision, D2 + CME) with conventional D2 lymphadenectomy for locally advanced gastric adenocarcinoma: study protocol for a randomized controlled trial
Source: Trials. 2018 Aug 9;19:432. doi: 10.1186/s13063-018-2790-5 (PMC6085680; doi:10.1186/s13063-018-2790-5)
Supplement: Supplementary file 3 — Instance of the scoring criterion for mesenteric excision in D2 + CME procedure. Mesenteric scoring including four parameters: trijunction point exposure, mesogastrium body, smooth plane of surgical bed after mesenteric resection, and the high tie ligation of vessels. Each parameter is scored as 2 (good), 1 (moderate), or 0 (poor). The parametric scores are summed to get the mesenteric score, then the mesenteric scores of all the dissected mesogastria are summed to get the total score. (PDF 179 kb) [file 13063_2018_2790_MOESM3_ESM.pdf]

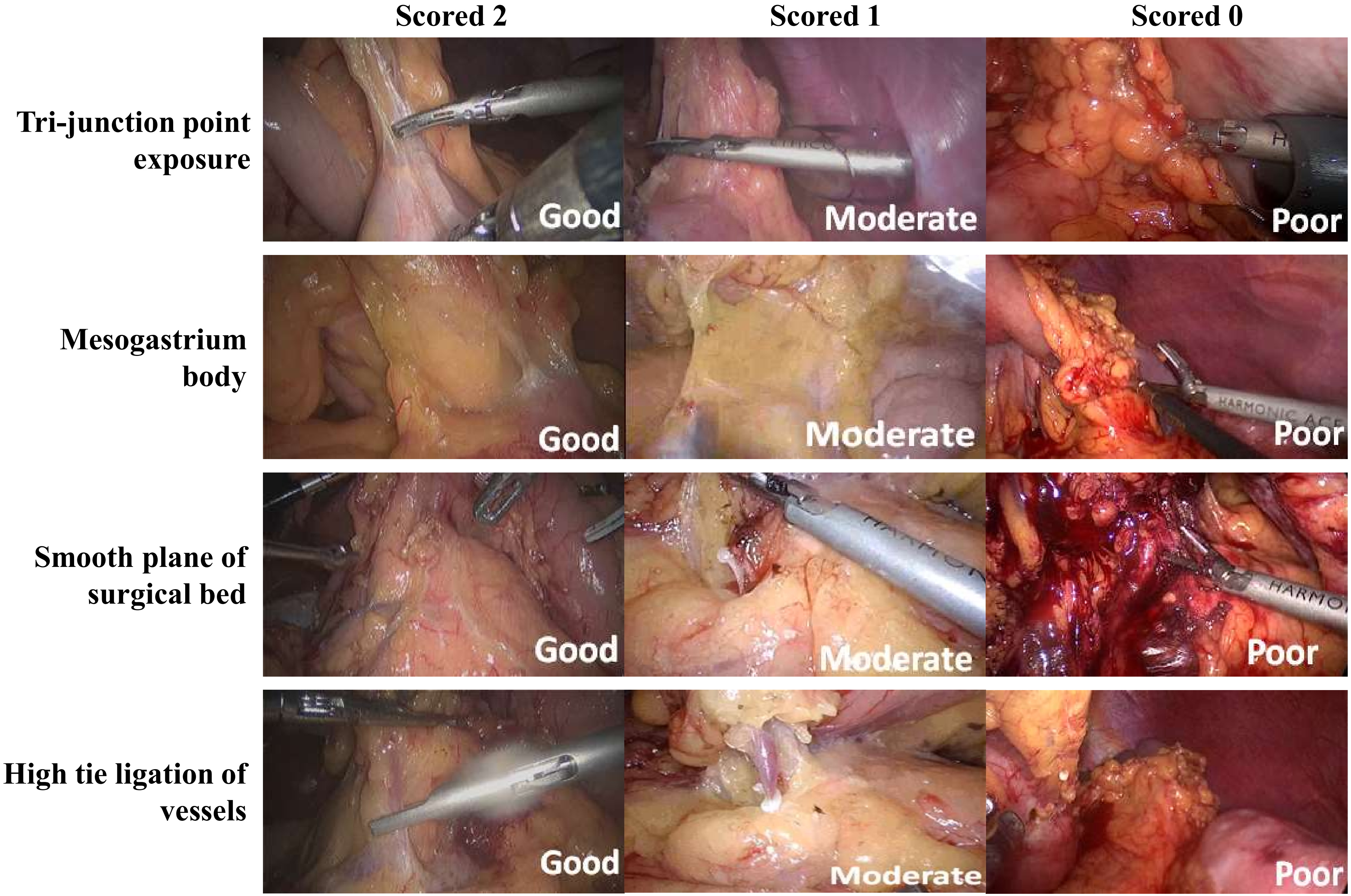
Scored 2
Scored 1
Scored 0
Tri-junction point exposure
Good
Moderate
Poor
Mesogastrium body
Good
Moderate
Poor
Smooth plane of surgical bed
Good
Moderate
Poor
High tie ligation of vessels
Good
Moderate
Poor
