# Supplementary material for: Prospective randomized controlled trial to compare laparoscopic distal gastrectomy (D2 lymphadenectomy plus complete mesogastrium excision, D2 + CME) with conventional D2 lymphadenectomy for locally advanced gastric adenocarcinoma: study protocol for a randomized controlled trial
Source: Trials. 2018 Aug 9;19:432. doi: 10.1186/s13063-018-2790-5 (PMC6085680; doi:10.1186/s13063-018-2790-5)
Supplement: Supplementary file 4 — Record table of mesenteric scoring in the D2 + CME procedure. (PDF 260 kb) [file 13063_2018_2790_MOESM4_ESM.pdf]

Evaluation Score Table of Mesenteric Excision

No.

Video Record ID: **Evaluation Date:**

1. Left Gastroepiploic Mesogastrium:

a) Tri-junction point exposure (2: Good; 1: Moderate; 0: Poor) Score:___
b) Mesogastrium body (2: Good; 1: Moderate; 0: Poor) Score:___
c) Smooth plane of surgical bed (2: Good; 1: Moderate; 0: Poor) Score:___
d) High tie ligation of vessels (2: Good; 1: Moderate; 0: Poor) Score:___

Mesenteric Score:_____

2. Right Gastroepiploic Mesogastrium:

a) Tri-junction point exposure (2: Good; 1: Moderate; 0: Poor) Score:___
b) Mesogastrium body (2: Good; 1: Moderate; 0: Poor) Score:___
c) Smooth plane of surgical bed (2: Good; 1: Moderate; 0: Poor) Score:___
d) High tie ligation of vessels (2: Good; 1: Moderate; 0: Poor) Score:___

Mesenteric Score:_____

3. Left Mesogastrium:

a) Tri-junction point exposure (2: Good; 1: Moderate; 0: Poor) Score:___
b) Mesogastrium body (2: Good; 1: Moderate; 0: Poor) Score:___
c) Smooth plane of surgical bed (2: Good; 1: Moderate; 0: Poor) Score:___
d) High tie ligation of vessels (2: Good; 1: Moderate; 0: Poor) Score:___

Mesenteric Score:_____

4. Right Mesogastrium:

a) Tri-junction point exposure (2: Good; 1: Moderate; 0: Poor) Score:___
b) Mesogastrium body (2: Good; 1: Moderate; 0: Poor) Score:___
c) Smooth plane of surgical bed (2: Good; 1: Moderate; 0: Poor) Score:___
d) High tie ligation of vessels (2: Good; 1: Moderate; 0: Poor) Score:___

Mesenteric Score:_____

5. Posterior Mesogastrium:

Mesenteric Score (2: Good; 1: Moderate; 0: Poor):_____

Total Score: **Qualified (Yes/No):**

Inspector Signature:
